# Diet Breadth Affects Bacterial Identity but Not Diversity in the Pollen Provisions of Closely Related Polylectic and Oligolectic Bees

**DOI:** 10.3390/insects11090645

**Published:** 2020-09-20

**Authors:** Jason A. Rothman, Diana L. Cox-Foster, Corey Andrikopoulos, Quinn S. McFrederick

**Affiliations:** 1 Department of Molecular Biology and Biochemistry, University of California, Irvine, CA 92697, USA; rothmanj@uci.edu; 2 Department of Entomology, University of California, Riverside, 900 University Avenue, Riverside, CA 92521, USA; 3 USDA-ARS Pollinating Insect-Biology, Management, and Systematics Research, Logan, UT 84322, USA; cja576@gmail.com; 4 Department of Biology, Utah State University, UMC5310, Logan, UT 84322, USA

**Keywords:** specialist, generalist, microbiome, foraging, flowers

## Abstract

**Simple Summary:**

Solitary bees are important pollinators in managed and wild ecosystems. Across the bee phylogeny, bees may forage on a single species of plant, few plant species, or a broad diversity of plants. During foraging, these bees are often exposed to microbes, and in turn, may inoculate the brood cell and pollen provision of their offspring with these microbes. It is becoming evident that pollen-associated microbes are important to bee health, but it is not known how diet breadth impacts bees’ exposure to microbes. In this study, we collected pollen provisions from the bees *Osmia lignaria* and *Osmia ribifloris* at four different sites, then characterized the bacterial populations within the pollen provisions with 16S rRNA gene sequencing. We found that diet breadth did not have large effects on the bacteria found in the pollen provisions. We also note that the bacterial communities were slightly different between bee species and site, and there was minimal overlap in the unique bacterial variants between sites and bee species too. Our research supports the hypothesis of environmental transmission for solitary bee microbes, and we suggest future studies investigate the impacts of microbes on larval health.

**Abstract:**

Mounting evidence suggests that microbes found in the pollen provisions of wild and solitary bees are important drivers of larval development. As these microbes are also known to be transmitted via the environment, most likely from flowers, the diet breadth of a bee may affect the diversity and identity of the microbes that occur in its pollen provisions. Here, we tested the hypothesis that, due to the importance of floral transmission of microbes, diet breadth affects pollen provision microbial community composition. We collected pollen provisions at four sites from the polylectic bee *Osmia lignaria* and the oligolectic bee *Osmia ribifloris.* We used high-throughput sequencing of the bacterial 16S rRNA gene to characterize the bacteria found in these provisions. We found minimal overlap in the specific bacterial variants in pollen provisions across the host species, even when the bees were constrained to foraging from the same flowers in cages at one site. Similarly, there was minimal overlap in the specific bacterial variants across sites, even within the same host species. Together, these findings highlight the importance of environmental transmission and host specific sorting influenced by diet breadth for microbes found in pollen provisions. Future studies addressing the functional consequences of this filtering, along with tests for differences between more species of oligoletic and polylectic bees will provide rich insights into the microbial ecology of solitary bees.

## 1. Introduction

Most bee species are considered mass provisioners—i.e., they build a brood cell into which they pack a mixture of pollen and nectar, deposit an egg on the pollen provision, and seal off the brood cell while the offspring develops [1]. This brood cell is left sealed until the fully developed bee emerges by breaking through the cell cap. While the mother bee (or possibly a sister bee in social species) creates the pollen provision for the developing bee, numerous other creatures may enter the brood cell. Organisms found in bee brood cells include—but are surely not limited to—nematodes [2], mites [3], springtails [4], bacteria [5], and fungi [6]. The bee brood cell can therefore be considered a miniature ecosystem [3], and how the interactions occurring within these tiny ecosystems affect bee health is a fascinating question.

High-throughput sequencing has allowed for detailed surveys of the diversity of the microbes that inhabit pollen provisions [7]. Early next-generation sequencing surveys of pollen provisions suggested that many bacteria found in pollen provisions may be acquired from flowers [8,9]. The observation that the same bacteria inhabit flowers, pollen provisions, and bee guts was subsequently verified [10]. Further studies then linked foraging to microbial transmission, which is more apparent when characterizing a network of plants, multiple bee species, and bacteria [11] than when studying a single population of bees [12]. When looking at multiple populations of one species across habitats, pollen usage and fungi co-vary more than pollen usage and bacteria [13]. The consensus arising from these studies is that flowers serve as transmission hubs for pollen-associated microbes, but the characteristics of pollen provisions may determine which microbes thrive there.

The importance of pollen-associated microbes on bee health is becoming evident. The genomes of pollen-associated lactobacilli contain genes involved in osmotic stress tolerance, detoxification of metals and other toxicants, and pollen wall degradation [14]. That these microbes exhibit genomic adaptations for rapid growth in nutrient-rich environments suggests that they likely ferment sugars found in pollen provisions and may exclude spoilage organisms, as their close relatives do in sourdough bread dough [15]. Experimental evidence for a nutritional role of pollen-borne microbes is also mounting. Isotopic signatures of diet suggest that bee larvae from a diversity of bee species are not truly herbivores as one would expect, but instead exhibit omnivorous or even carnivorous traits [16]. This finding suggests that bee larvae are consuming microbes in their pollen provisions. Feeding bee larvae different ratios of sterilized to normal pollen leads to differences in growth rates and survival, again suggesting that larvae consume pollen-borne microbes [17]. Similarly, whether microbes were present or absent in pollen had a greater influence on larval development compared to whether the pollen was collected by con-specific or different bee species for larvae of the specialist blueberry pollinator *Osmia ribifloris* [18]. Altogether, these studies are beginning to illustrate the importance of microbes in the pollen provisions of wild and solitary bees for larval health.

One open question in the study of the microbiome of the pollination landscape is how diet breadth affects exposure to and acquisition of microbes. Across the bee phylogeny, there is a diversity of diet breadths, with some bees visiting a broad diversity of plant species (polylectic or generalist bees) and others visiting a limited number of plants (oligolectic or specialist bees), or even a single plant (monolectic bees), with gradations in between these groups [19]. As specialist bees visit fewer plants, they may acquire a distinct microbial community compared to generalist bees. Conversely, if specialists interact with the same plants as generalists do, both classes of bees may be exposed to the same microbes. The microhabitats of specialist and generalist bee pollen provisions may filter different microbes based on pollen and nectar chemistry, altering microbial composition as has been found with nectar microbial communities [20]. Comparing the pollen-provision microbial communities of closely related specialist and generalist bees will help address how diet breadth affects microbe exposure and establishment.

To help understand how diet breadth affects bee nest microbial communities, we characterized the pollen provision microbial communities of a pair of closely related bees collected across four sites: *Osmia lignaria,* a polylectic bee that prefers to forage on orchard trees in the family Roseaceae [21]; and *O. ribifloris,* a specialist on blueberries and its relatives with *Berberis* serving as an alternate host [22]. We address the hypothesis that, due to the importance of floral transmission for microbes found in the pollen provisions of megachilid bees [10], diet breadth affects pollen provision microbial composition. We predicted that the specialist bee would harbor fewer species of microbes and show less variation across sites compared to the generalist bee.

## 2. Materials and Methods

### 2.1. Sample Collection and Locations

We collected pollen provisions from the nest cells of two bee species native to the area of Logan, Utah, USA: *Osmia lignaria propinqua* and *Osmia ribifloris biedermannii* in Spring 2015. We chose four locations near Logan that varied in floral composition and interaction with other bee species: Site 1 was a cultivated floral garden located in a suburban area (41°45′05″ N, 111°47′30″ W) with a combination of native and introduced floral species, including *Rubus idaeus* L. (American red raspberry) and *Calluna vulgaris* (Scotch heather). Site 2 was a bee-tight screenhouse (dimensions: 14 × 9 × 3 m) (41°45′29″ N, 111°48′44″ W) that contained older, in-ground plantings of *R. idaeus* and *Berberis fremontii* (Fremont’s mahonia), along with potted *C. vulgaris*. Sites 3 and 4 were natural areas of Logan Canyon (41°47′55″ N, 111°39′04″ W at 1671 m elevation; 41°48′15″ N, 111°37′49″ W at 1665 m elevation, respectively) that contained limestone cliffs and a mixed forest, and were approximately 1836 m apart, which is likely outside of the flight range of *Osmia* species [23]. We note that the locations differed in the composition of their pollinator communities: Site 1 had a diverse pollinator community present, including numerous honey bee and bumble bee colonies. Site 2 was a research screenhouse that had no honey bees nor bumble bees present for at least five preceding years. Sites 3 and 4 had diverse pollinator communities without honey bees present, and we did not collect any *O. ribifloris* from Site 4. See supplemental Table S1 for the number of each bee species collected at each site. There were also differences in the sources of sampled *Osmia* nests between locations: We sampled natural *Osmia* populations from Sites 1, 3, and 4, while we sampled nests made by commercially acquired bees for Site 2. In this location, *O. lignaria* were sourced from Watts Solitary Bees (Bothell, WA, USA) and *O. ribifloris* were purchased from NativeBees.com (Kaysville, UT, USA).

Within each location, we sampled the pollen provisions from cavities (7 mm × 14 cm) within wooden nesting blocks that were lined with paper straws and placed in each location for one week. As both species of bee will nest in these cavities at the same time, we were able to concurrently sample newly made nests. We used 1–6 nesting straws per bee species, then removed the straws from the nest blocks, numbered them sequentially, and X-rayed the straws to confirm that eggs were present and had not yet hatched. Once the straws were collected, we carefully slit open the straws, removed the pollen provisions from each initially formed cells (positions 1–4), then sterilely removed the eggs from nest cells before DNA extraction. We carefully excluded cell wall partitions from the collection of the pollen provision. Each cell was treated as a separate sample.

### 2.2. DNA Extraction and Library Preparation

We extracted DNA from the pollen provisions based on a modified protocol from Engel et al. 2013 [24], Rothman et al. [25], and Pennington et al. 2018 [26]. We used a Qiagen DNeasy Blood and Tissue kit (Qiagen, Valencia, CA, USA) for the DNA extractions, with slight modifications. We sterilely transferred entire pollen provisions to a 96-well tissue lysis plate (Qiagen, Valencia, CA, USA), then added 100 µL glass beads, one 3.2 mm steel-chrome bead, and 180 µL buffer ATL to each well. We bead-beat the mixture at 30 Hz for three minutes, turned the plates over, then bead-beat for three more minutes. We incubated the mixture at 50 °C overnight, then followed the rest of the manufacturer’s protocol to finish the DNA extraction.

We used the extracted DNA to prepare 16S rRNA gene libraries as in McFrederick and Rehan 2016 [12], Rothman et al. 2019 [27], and Pennington et al. 2017 [28]. Briefly, we used a dual-indexing approach to build an amplicon construct consisting of the universal primers 799F-mod3 [29] and 1115R [30] (which reduces plant plastid contamination while allowing bacterial amplification), a unique 8-mer barcode, and the Illumina adapter sequence as in Hanshew et al. 2013 [29]. We built the libraries in two rounds of PCR amplification: First, we used 4 µL of template DNA, 0.5 µL of 10 µM forward and reverse barcoded primers, 10 µL water, and 10 µL of 2× Pfusion DNA polymerase (New England Biolabs, Ipswich, MA, USA) with an annealing temperature of 52 °C for 30 cycles. Next, we cleaned the PCR product with a MoBio UltraClean PCR cleanup kit (Invitrogen, Carlsbad, CA, USA). We then used the cleaned amplicons as template for another PCR reaction, with the following conditions: 1 µL template DNA, 0.5 µL of 10 µM primers PCR2F and PCR2R [12], 13 µL water, and 10 µL 2× Pfusion DNA polymerase with an annealing temperature of 58 °C for 15 cycles. We cleaned and normalized the libraries with a SequalPrep Normalization kit (ThermoFisher, Waltham, MA, USA), pooled 5 µL of each library, then cleaned and concentrated the libraries with a MoBio UltraClean PCR cleanup kit (Invitrogen, Carlsbad, CA, USA). Lastly, we used an Illumina MiSeq to sequence the libraries at 2 × 300 cycles in the UC Riverside Genomics Core Facility.

### 2.3. Bioinformatics and Statistics

We processed our 16S rRNA libraries with QIIME2-2019.7 [31]. We trimmed adapters, sequencing primers, and low-quality ends off the sequences with QIIME2, then used DADA2 [32] to remove chimeras, cluster reads into Amplicon Sequence Variants (ASVs; 16S rRNA gene sequences with 100% matched identities), and discard singletons. We assigned taxonomy to the reads with the q2-feature-classifier [33], using the SILVA database as a reference [34], and confirmed taxonomic assignment with BLAST to both NCBI nt/nr and 16S Microbial databases. Next, we removed features corresponding to mitochondria, chloroplast, or contaminants as identified in our reagent control blank samples [35], and validated with the R package “decontam.” We then aligned the 16S rRNA gene sequences with MAFFT [36], and used FastTree to generate phylogenies of the reads [37]. We then tabulated the ASVs (File S1) and used this table for rarefaction analyses and to calculate Shannon Diversity Indices, Bray–Curtis Dissimilarities, and both weighted and unweighted UniFrac distances for the samples. Additionally, we calculated diversity metrics on the ASV table after removing ASVs at less than 0.01% overall abundance. We tested the alpha diversity of our samples for statistical significance with Kruskal–Wallis tests, the beta diversity through the Adonis PERMANOVA (ANOVA with 999 permutations) and Levene’s test for heterogenous dispersion in R 3.5.1 [38] with the package “vegan” [39], analyzed differential abundance of bacterial families with ANCOM [40], and corrected *p* values for multiple comparisons with the Benjamini–Hochberg method where appropriate. Lastly, we visualized our data with “ggplot2” [41], “metacoder” [42], and the BEG Venn diagram tool (http://bioinformatics.psb.ugent.be/webtools/Venn/).

Raw sequencing data are available on the NCBI Sequence Read Archive under BioProject accession number PRJNA646828.

## 3. Results

### 3.1. Alpha Diversity and Library Statistics

**Figure 1:**
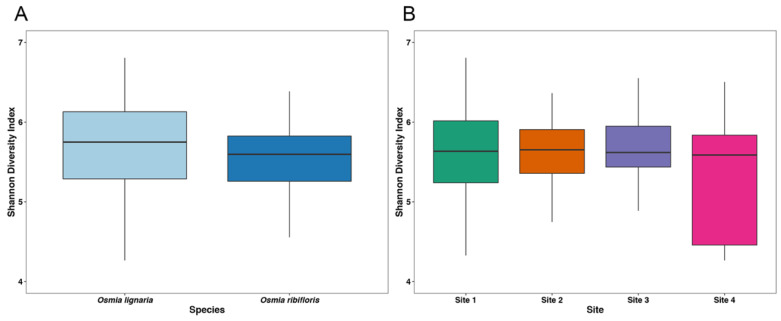
Boxplots of alpha diversity in (**A**) *Osmia* species, and (**B**) sampling site as measured by the Shannon Diversity Index. Alpha diversity was not significantly different between *Osmia* species (H = 1.76, *p* = 0.18) or sampling site overall (H = 1.42, *p* = 0.70) and pairwise between sites (*p*_adj_ > 0.05 for each comparison).

Through Illumina MiSeq sequencing, we obtained 207,650 quality-filtered 16S rRNA gene reads with an average of 2186 reads per sample (N = 92 samples, 3 reagent control blanks) which clustered into 4762 unique ASVs. Through rarefaction analyses (Figure S1), we determined that we captured adequate diversity coverage through a sequencing depth of 799 reads per sample. This rarefaction depth resulted in the loss of 7 samples (N = 85). We analyzed the alpha diversity (as measured through the Shannon diversity index) of our samples through Kruskal–Wallis testing, and did not find any significant effect of bee species (H = 1.76, *p* = 0.18) or sampling site overall (H = 1.42, *p* = 0.70) or pairwise between sites (*p*_adj_ > 0.05 for each, Table S2) on ASV diversity (Figure 1).

### 3.2. Taxonomic Description of the Data

**Figure 2:**
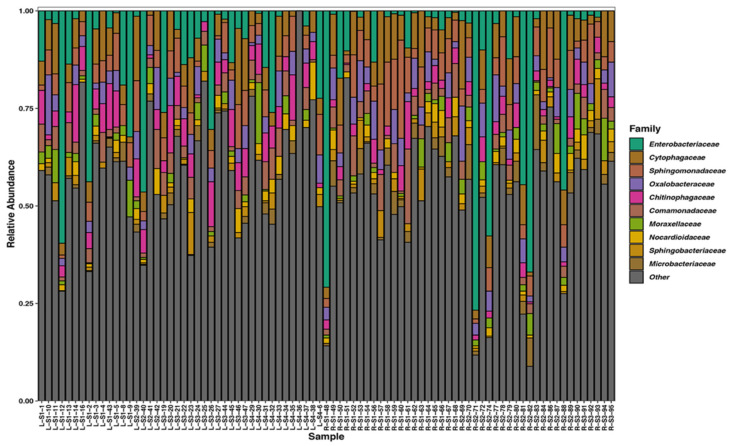
Stacked bar plot of the average relative abundance of the ten most proportionally abundant bacterial families across all samples and all other families combined as “other”. Sample names contain identifying information about species and site: “L” or “R” corresponds to *O. lignaria* and *O. ribifloris* respectively, “S#” corresponds to sites 1–4, and the last number is the unique sample number. Bacterial family is denoted by color.

**Figure 3:**
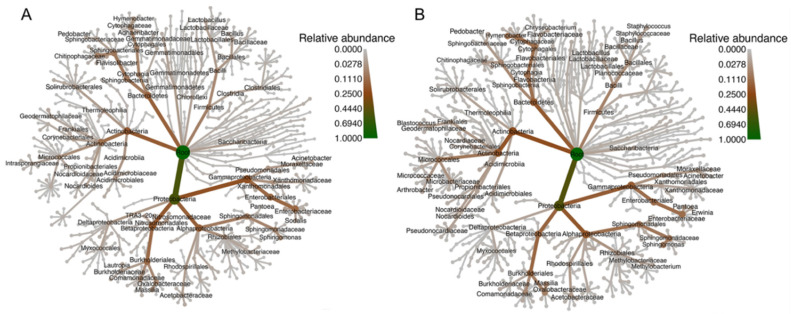
“Heat trees” indicating the hierarchical phylogenetic relationships and relative abundances of taxa within (**A**) *O. lignaria* and (**B**) *O. ribifloris*. Branch color denotes relative abundance of the taxonomic rank within all samples from within each species, and taxon labels represent the 75 most proportionally abundant taxa.

We analyzed the bacterial composition of our samples and found that the ten most proportionally abundant families in our samples regardless of site were as follows: *Enterobacteriaceae* (13.6%), *Cytophagaceae* (6.5%), *Sphingomonadaceae* (6.0%), *Oxalobacteraceae* (4.7%), *Chitinophagaceae* (4.4%), *Comamonadaceae* (3.6%), *Moraxellaceae* (2.2%), *Nocardioidaceae* (2.0%), *Microbacteriaceae* (1.9%), and *Sphingobacteriaceae* (1.8%) (Figure 2). We also establish that ASVs belonging to the genera *Pantoea* (8.4%), *Sphingomonas* (5.6%), and *Hymenobacter* (4.1%) were the most proportionally abundant and plotted “heat trees” of the microbial community of our samples to show all taxonomic ranks present in both bee species (Figure 3).

### 3.3. Shared and Unique Amplicon Sequence Variants by Bee Species and Site

**Figure 4:**
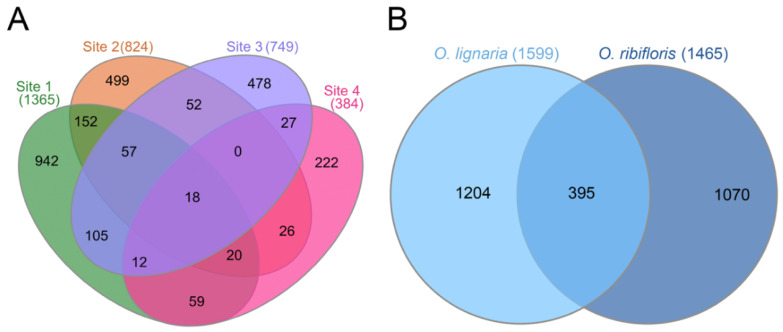
Venn diagrams of Amplicon Sequence Variants (ASVs) in greater than 0.01% overall abundance found in (**A**) sampling locations between all species with color denoting site and (**B**) ASVs in greater than 0.01% overall abundance found in species between all sampling locations with color denoting species. Numbers in both panels denote number of ASVs present in each location/species or the union between multiple categories.

As our samples contained a wide diversity of bacterial taxa, we wanted to determine the impact of sampling site on the presence or absence of individual ASVs. As ASVs in very low abundance are likely not biologically relevant and may represent sequencing and/or classification errors, we compared the presence/absence of ASVs at greater than 0.01% overall abundance only. Of the 2669 unique bacterial ASVs, only 18 (0.67% of ASVs) were present in all four sites, and 2141 were unique to individual sites (80.2% of ASVs, Figure 4). Notably, the 18 “cosmopolitan” ASVs corresponded to four ASVs of *Acinetobacter*, three ASVs of *Hymenobacter*, two ASVs of *Lautropia*, and one ASV of *Chryseolinea*, *Flavitalea*, *Illumatobacter*, *Marmoricola*, *Massilia*, *Methylobacterium*, *Pantoea*, *Ralstonia*, and *Sphingomonas*. We analyzed our data in a similar fashion specifically looking at host bee species and found that ASVs were mostly unique to each species, with *O. lignaria* having 1204 ASVs (45.1%) solely, *O. ribifloris* having 1070 ASVs (40.1%) solely, and both species sharing 395 ASVs (14.8%; Figure 4), and we report the ASVs corresponding to each bee species broken down by site in supplemental Figure S2. We also note that no ASVs were present in greater than 50% of samples within each species, so there are likely no taxa “core” to the microbiome of *O. lignaria* or *O. ribifloris* pollen provisions.

### 3.4. Beta Diversity of Microbes within Osmia Species and Site Location, and Differential Abundance of Bacterial Families

**Figure 5:**
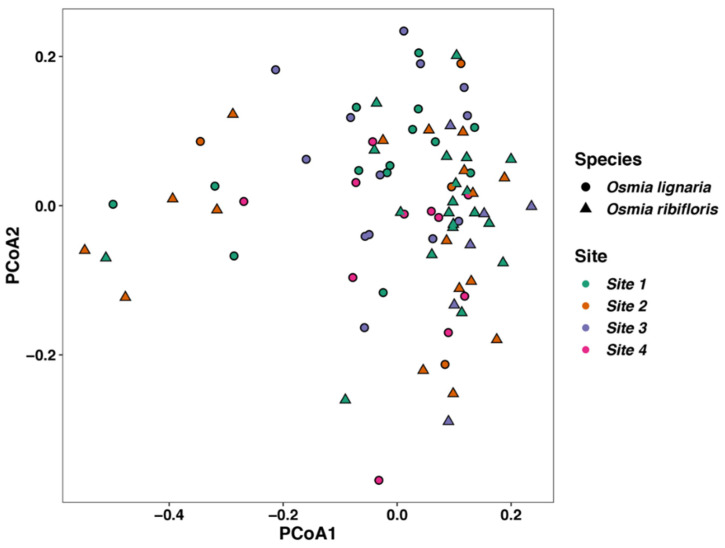
Principal components analysis (PCoA) ordination of the weighted UniFrac distances of microbial communities of both bee species (*Osmia lignaria* and *Osmia ribifloris*) and sampling site. There was a significant difference in the weighted UniFrac distances between bee species (F = 3.25, R^2^ = 0.037, *p* = 0.004) and geographic location (F = 1.58, R^2^ = 0.054, *p* = 0.03). Point shape denotes bee species, and color denotes site.

We tested for significant differences in beta diversity with Adonis PERMANOVA and found that there was a significant effect of bee species by Bray–Curtis (F = 1.23, R^2^ = 0.015, *p* = 0.006), unweighted UniFrac (F = 1.95, R^2^ = 0.022, *p* < 0.001), and weighted UniFrac matrices (F = 3.25, R^2^ = 0.037, *p* = 0.004). We also establish that sampling site had a significant effect on the beta diversity of our samples for Bray–Curtis (F = 1.11, R^2^ = 0.040, *p* < 0.001), unweighted (F = 1.44, R^2^ = 0.05, *p* < 0.001), and weighted UniFrac (F = 1.58, R^2^ = 0.054, *p* = 0.03), with no interaction of bee species and sampling site (*p* = 0.50, 0.45, and 0.44 respectively). Lastly, we used a nested design in Adonis to analyze the beta diversity of the individual straws in which the bees built their nests in at each site. We found no significant effect of straw within site for Bray–Curtis dissimilarities, and unweighted or weighted UniFrac distances (*p* = 0.09, 0.07, and 0.08, respectively). We also ran Adonis tests as above on distance matrices generated from the ASV table with ASVs at <0.01% overall abundance and a rarefaction depth of 501. We did not find any meaningful statistical differences between these analyses and our analyses with the full ASV table, and we report those statistics in supplemental Table S3. Likewise, due to there being no *O. ribifloris* present at Site 4, we ran diversity analyses on Sites 1–3 only, but our statistical interpretation of the data remained the same (Adonis test for species: [Bray–Curtis, *p* = 0.045; unweighted UniFrac, *p* < 0.001; weighted UniFrac, *p* = 0.004], Adonis test for site: [Bray–Curtis, *p* = 0.007; unweighted UniFrac, *p* = 0.005; weighted UniFrac, *p* = 0.041], Adonis test for species x site interaction [Bray–Curtis, *p* = 0.50; unweighted UniFrac, *p* = 0.36; weighted UniFrac, *p* = 0.42]). We also did not find that our data was heterogeneously dispersed for either site or species for any of the distance matrix metrics (*p* > 0.05). Lastly, we performed a Principal Coordinates Analysis (PCoA) and plotted the ordination of the weighted UniFrac distances of our samples (Figure 5).

**Figure 6:**
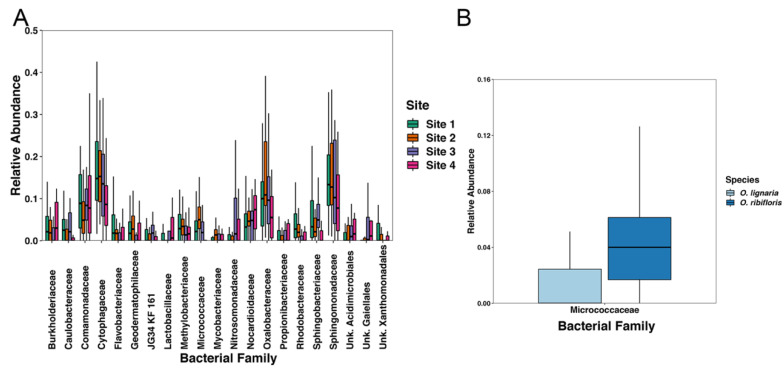
Boxplots of the average relative abundances of bacterial families (present at greater than 0.5% relative abundance) that were significantly differentially abundant as measured by ANCOM (Wald > 1 for each family) between (**A**) sampling locations and (**B**) *Osmia* species. Whiskers represent 1.5× the interquartile range of the data.

We used ANCOM to compare bacterial families in greater than 0.5% proportional abundance between all of our sample sites within both species of bee. We found the following significantly differentially abundant families (Wald > 1, Table S4): *Nitrosomonadaceae, Micrococcaceae, Rhodobacteracea, Sphingomonadaceae, Nocardioidaceae, Sphingobacteriaceae, Caulobacteraceae, Lactobacillaceae, Cytophagaceae, Oxalobacteraceae, Methylobacteriaceae, Geodermatophilaceae, Flavobacteriaceae, Comamonadaceae,* JG34-KF-161 (Order: Sphingomonadales), *Propionibacteriaceae, Mycobacteriaceae, Burkholderiaceae*, and uncultured families within the orders Gaiellales, Acidimicrobiales, and Xanthomonadales (Figure 5). We also compared bacterial families between bee species and found that only *Micrococcaceae* was significantly more proportionally abundant in *O. ribifloris* (Figure 6, Table S4).

## 4. Discussion

Contrary to our predictions, diet breadth did not have large effects on the bacterial communities found in the pollen provisions of two closely related bee species. The number of different bacterial variants found in pollen provisions did not differ between the oligolectic *O. ribifloris* and the polylectic *O. lignaria.* Likewise, the pollen provisions of both species harbored subtly different microbial communities across sites when relatedness of bacteria is considered. At the sequence variant level, however, we detected minimal overlap in bacteria across sites—even between nests from the same species—suggesting that the local environment largely determines which microbes are recruited into pollen provisions. Furthermore, no bacteria were constant enough to be considered as ‘core’ *Osmia* pollen provision bacteria, even at a minimal threshold of occurring in 25% of all samples. On the other hand, the two hosts—even when artificially restricted to foraging on only two plant species in the same cages—harbored different bacterial taxa in their pollen provisions. Surprisingly, only 18 bacterial variants were found at all four sites. Together, these results suggest that the different diet breadth of these two closely related species does not affect the microbial diversity of their provisions, but does appear to affect the specific variants of pollen-provision inhabiting microbes. Another possible source of bacterial community variation between the two species could be the different materials with which these two *Osmia* species partition their nests. *Osmia ribifloris* uses masticated leaves [22] while *O. lignaria* uses mud [21] to partition the brood cells in their nests. As we have previously reported in our study of the alfalfa leafcutting bee [43], these nest components may affect the microbial community in orchard bee nests and should be the focus of future research.

Other studies have found that bee species identity, foraging patterns, and geography can affect the microbes found in pollen provisions. McFrederick and Rehan [13] showed that the pollen provision microbiome of *Ceratina calcarata* co-varies with pollen usage across habitats, especially when considering fungi. Voulgari-Kokota et al. [11] showed that bee species and foraging patterns drive the bacterial communities found in pollen provisions. Our findings add to the consensus of these other studies that floral transmission and pollen usage influence the composition of the pollen provision microbiome. Our study also extends these previous studies by adding an understanding of how diet breadth does (microbial identity) and does not (microbial diversity) affect the pollen provision microbial community in these two species of *Osmia*. Whether the different bacterial variants found in the host species and sites across studies is due solely from different transmission networks, filtering of microbes via differential floral and pollen provision chemistry, or a combination of both requires further study.

As in other bee species’ pollen provisions, *Osmia* spp. pollen provisions contain a wide diversity of microbes. In agreement with these previous studies, the bacteria identified here can also be found in the environment, in both flowers and soil. For example, four of the 18 ASVs that were found at all sites in our study belonged to the genus *Acinetobacter*. This genus is ubiquitous in the environment and has been found in association with plants and animals, in floral nectar, in sewage, and in water and soil [44]. The small fragment of the 16S rRNA gene that we use for Illumina sequencing rarely allows species-level resolution of these bacteria, so we are unable to determine whether these taxa were sourced from the mud that the bees used to partition their brood cells or from the nectar they mixed into their pollen provisions. *Acinetobacter*, however, has been reported in association with pollen provisions of several different bee species [9,10,11,12], and it is therefore not surprising that it occurs at all study sites.

One conspicuous group of bacteria that are found in many pollen provision microbiomes [8,9,10,11,12,13,43,45] but were uncommon here are bacteria from the *Apilactobacillus micheneri* clade [46]. These bacteria have been found in the pollen provisions of other megachilid species in North America and Europe [10,11,43,47], but it is becoming clear that they are unevenly distributed across species [11,47]. For example, in Germany, *Apilactobacillus* spp. are abundant in the pollen provisions of *Megachile* spp., at variable abundances in *O. caerulescens* provisions, at low abundance in *Heriades truncorum* provisions, and absent in *O. bicornis* and *O. leaiana* provisions [11,47]. In Texas (USA), we detected these same lactobacilli at high relative abundances in pollen provisions of *Osmia chalybea, Osmia subfasciata* and *Megachile policaris* [10]. Here, we report that lactobacilli are present only at very low relative abundances in the pollen provisions of *O. lignaria* and *O. ribifloris,* and that the *A. micheneri* clade lactobacilli are absent. As these bacteria have been isolated from flowers in both Texas and California [10,14], we hypothesize that either foraging preferences or pollen provision chemistry drives the presence or absence of these lactobacilli, and understanding the apparently cosmopolitan phenomenon of uneven distribution of lactobacilli across wild and solitary bee species should be a priority for pollen provision microbial community studies.

Many of the bacteria that we identified in *O. lignaria* pollen provisions have also been found in association with *O. lignaria* adults [48]. Cohen et al. [48] found that adults that had been foraging in the environment had a different or more variable microbiome compared to bees that emerged under sterile conditions in the lab, again supporting the importance of environmental transmission for the wild and solitary bee microbiome. Many of the bacteria found in adult *O. lignaria* adults are found in the environment and in the pollen provisions that we studied here. For example, *Massilia* is a root-colonizing soil bacterium [49] that has been found in *O. bicornis* nests [9]. This bacterium may be found in adult *O. lignaria* and their pollen provisions due to the adult’s habit of collecting mud to build partitions between brood cells in their nests [50]. *Pantoea* is a plant-associated microbe that is abundant in the environment but has also been reported in association with other solitary bees [43,51] and honey bees [52]. Surprisingly, Cohen et al. [48] found lactobacilli associated with adult *O. lignaria,* and additionally found that the abundance of flowers at a site positively correlated with the relative abundance of lactobacilli associated with adult *O. lignaria.* Future studies examining the adult and pollen provision microbiome of *O. lignaria* will help unravel how the environment shapes the microbiome of these separate but connected niches.

The sole bacterial family that we found to be differentially abundant by host species was *Micrococcacea*. *Micrococcacea* is a diverse family that occurs in the environment, includes a commensal but opportunistic human pathogen [53], and has been classified as a pathogen honey bees [54]. As *Micrococcacea* represented around 4% of the reads in seemingly healthy *O. ribofloris* nests, it is unlikely that this bacterium is pathogenic to *Osmia.* The differential abundance between the two *Osmia* species may mean that it is somehow important for bee health, but may also be due to the differential abundance between sampling site too. Along this thought, we found that many bacterial families were differentially abundant between geographical location. This provides further support to the hypothesis of environmental and floral transmission for solitary bee microbes as has been shown in previous studies [10,11,13].

## 5. Conclusions

Our study suggests that diet breadth may be important for the identity, but not the diversity, of bacteria that are found in solitary bee pollen provisions. We note, however, that as we only test a single oligoectic and a single polylectic species, studies with greater species-level replication are still needed. As mounting evidence suggests that pollen-provision microbes are important for bee health [14,16,17,18], future studies should address the potential functions and genomic capabilities of *O. ribifloris* and *O. lignaria* associated bacteria. Of special interest is the minimal overlap in specific bacterial variants across sites and host species. This lack of congruence highlights the importance of environmental transmission for these two bee species, yet differential filtering of bacteria by host species even when those hosts artificially share the same forage in cages. How this environmental transmission and microbial filtering affect larval health is the next pressing question to be answered.

## Supplementary Materials

The following are available online at https://www.mdpi.com/2075-4450/11/9/645/s1: Figure S1: Rarefaction analyses for each sample; Figure S2: Unique and shared ASVs for each bee species separated by sampling site. A–C corresponds to Site 1–3 respectively. Site 4 did not have any *Osmia ribifloris* present, so it is not shown here; Table S1: Sample sizes of *Osmia* spp. collected at each sampling site; Table S2: Alpha diversity statistics for the microbial communities based on species and site comparisons; Table S3: Beta diversity statistics of data with ASVs at <0.01% overall abundance removed, and a sampling depth of 501 reads per sample; Table S4: Wald scores of significantly differentially abundant bacterial families as analyzed by ANCOM between sites or bee species; File S1: Amplicon sequence variant (ASV) table with counts per sample, top BLAST hit, and SILVA taxonomic classification for all samples.
