# Supplementary material for: Diet Breadth Affects Bacterial Identity but Not Diversity in the Pollen Provisions of Closely Related Polylectic and Oligolectic Bees
Source: Insects. 2020 Sep 20;11(9):645. doi: 10.3390/insects11090645 (PMC7564857; doi:10.3390/insects11090645)
Supplement: Supplementary file 1 [file insects-11-00645-s001.zip › insects-897106-supple.pdf]

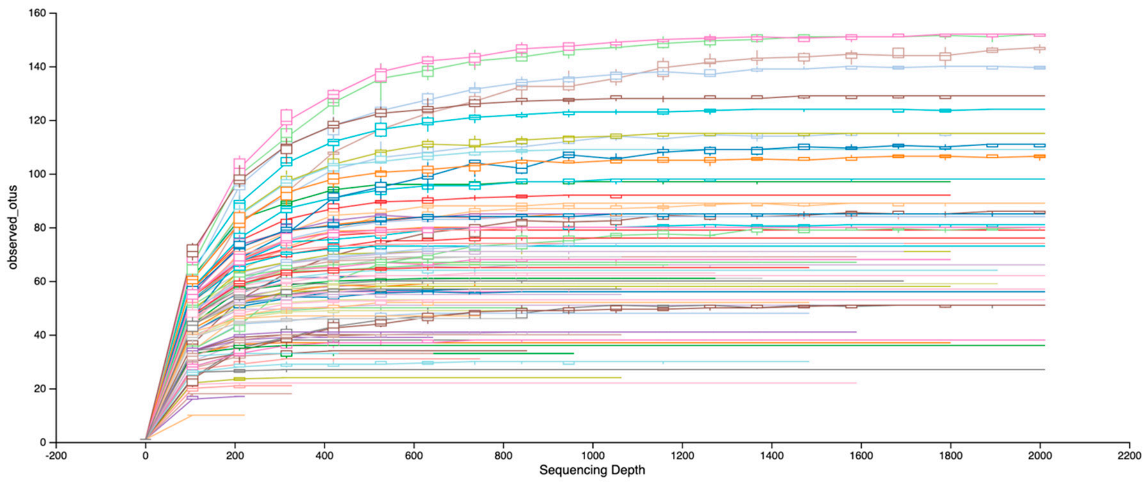

Figure S1. Rarefaction analyses for each sample. We obtained representative sequencing coverage at a read depth of 799 reads per sample.

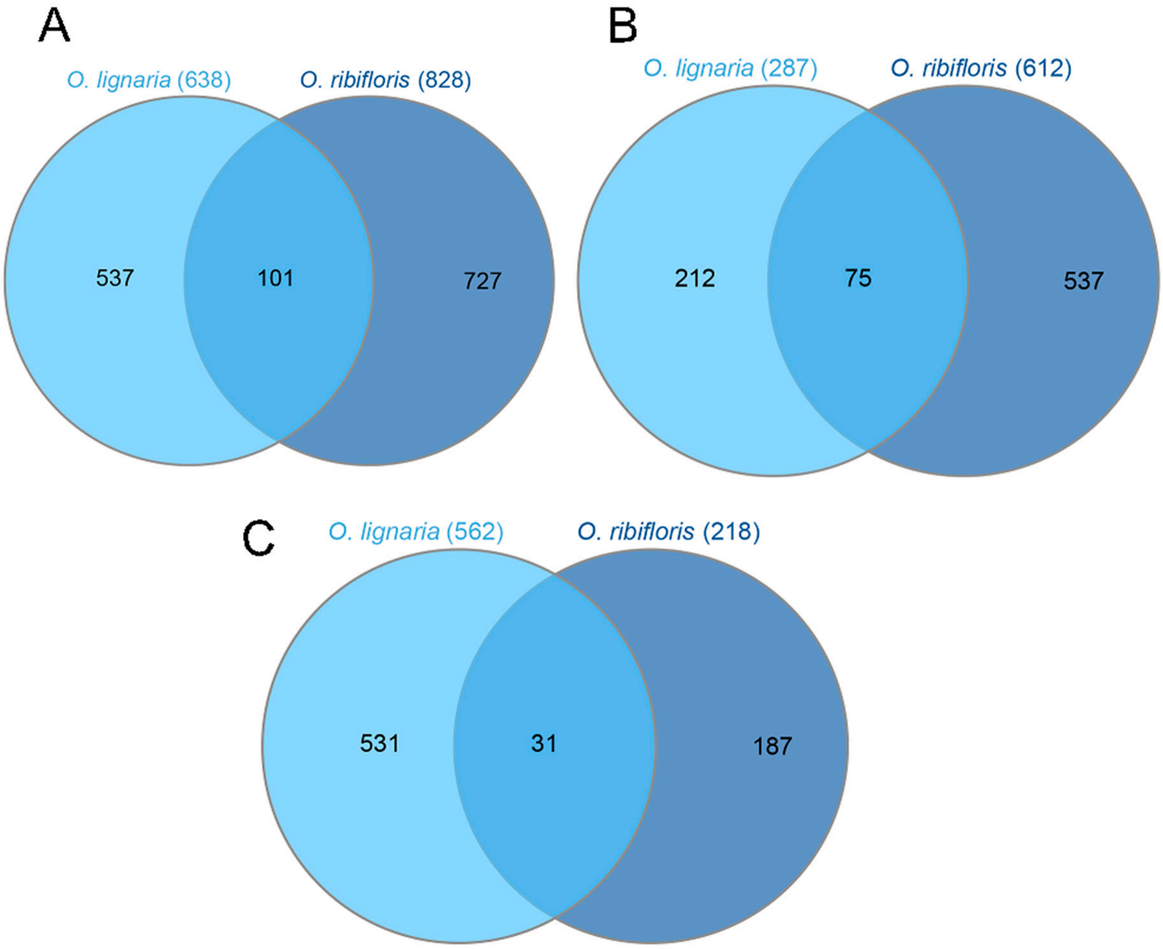

Figure S2. Unique and shared ASVs at greater than 0.01% overall abundance for each bee species separated by sampling site. Panels A–C corresponds to Site 1–3 respectively. Site 4 did not have any *Osmia ribifloris* present, so it is not shown here.

Table S1. Sample sizes of *Osmia* spp. collected at each sampling site.

Site	*Osmia lignaria* Collected	*Osmia ribifloris* Collected
Site 1	16	21
Site 2	4	21
Site 3	15	6
Site 4	12	0

Table S2. Alpha diversity statistics for the microbial communities based on species and site comparisons with the full ASV table and a sampling depth of 799 reads per sample. When removing ASVs at <0.01% abundance and using a read depth of 501, alpha diversity was still not significantly different by bee species (H = 0.92, p = 0.34), sampling site overall (H = 2.22, p = 0.53), or pairwise between sites (p_{adj} > 0.05 for each).

Categorical Comparison	Shannon (α Diversity)	
	H	p_{adj}
Osmia ribifloris vs *Osmia lignaria*	1.76	0.18
All Sites Overall	1.42	0.70
Site 1 vs Site 2	0.22	0.78
Site 1 vs Site 3	0.02	0.88
Site 1 vs Site 4	1.29	0.78
Site 2 vs Site 3	0.31	0.78
Site 2 vs Site 4	0.21	0.78
Site 3 vs Site 4	1.02	0.78

Table S3. Beta diversity statistics of data with ASVs at <0.01% overall abundance removed, and a sampling depth of 501 reads per sample.

Comparison	Bray-Curtis		Unweighted UniFrac		Weighted UniFrac	
	F	*p*	F	*p*	F	*p*
Species	1.25	0.005	2.04	0.002	3.18	0.01
Site	1.12	0.018	1.39	0.003	1.48	0.042
Species x Site	1.00	0.45	1.05	0.36	1.11	0.31
Straw within Site	1.02	0.23	1.01	0.46	1.12	0.27

Table S4. Wald scores of significantly differentially abundant bacterial families as analyzed by ANCOM between sites or bee species.

Comparison Across All Sites		Comparison Across Bee Species	
**Bacterial Family**	**Wald Score**	**Bacterial Family**	**Wald Score**
Unknown family, order Gaiellales	11	Micrococcaceae	18
Nitrosomonadaceae	11		
Micrococcaceae	9		
Rhodobacteraceae	8		
Sphingomonadaceae	6		
Nocardioidaceae	5		
Sphingobacteriaceae	4		
Caulobacteraceae	3		
Lactobacillaceae	3		
Unknown family, order Acidimicrobiales	3		
Cytophagaceae	2		
Oxalobacteraceae	2		
Unknown family, order Xanthomonadales	2		
Methylobacteriaceae	2		
Geodermatophilaceae	2		
Flavobacteriaceae	2		
Comamonadaceae	1		
JG34-KF-161, order Sphingomonadales	1		
Propionibacteriaceae	1		

Mycobacteriaceae	1
Burkholderiaceae	1
